# A novel vaccine candidate based on chimeric virus-like particle displaying multiple conserved epitope peptides induced neutralizing antibodies against EBV infection

**DOI:** 10.7150/thno.42494

**Published:** 2020-04-27

**Authors:** Xiao Zhang, Bingchun Zhao, Mingmei Ding, Shuo Song, Yinfeng Kang, Yang Yu, Miao Xu, Tong Xiang, Ling Gao, Qisheng Feng, Qinjian Zhao, Mu-Sheng Zeng, Claude Krummenacher, Yi-Xin Zeng

**Affiliations:** 1 State Key Laboratory of Oncology in South China, Collaborative Innovation Center for Cancer Medicine, Guangdong Key Laboratory of Nasopharyngeal Carcinoma Diagnosis and Therapy, Department of Experimental Research, Sun Yat-sen University Cancer Center, Sun Yat-sen University, Guangzhou, Guangdong, PR China; 2 State Key Laboratory of Molecular Vaccinology and Molecular Diagnostics, National Institute of Diagnostics and Vaccine Development in Infectious Diseases, School of Public Health, Xiamen University, Xiamen, Fujian, PR China; 3 Department of Biological Sciences and Department of Molecular and Cellular Biosciences, Rowan University, Glassboro, NJ, United States; 4 Present address: Vaccine Research Center, National Institute of Allergy and Infection Diseases, National Institutes of Health, Bethesda, MD, United States; 5 School of Medicine, Sun Yat-Sen University, Guangzhou, Guangdong, PR China; 6 State Key Laboratory of Molecular Vaccinology and Molecular Diagnostics, School of Life Sciences, Xiamen University, Xiamen, China

**Keywords:** Epstein-Barr virus, Envelope glycoprotein, gp350, Conserved epitope peptide, HBc149, Chimeric virus-like particle, Neutralizing antibody.

## Abstract

**Rationale**: Epstein-Barr virus (EBV) is the causative pathogen for infectious mononucleosis and many kinds of malignancies including several lymphomas such as Hodgkin's lymphoma, Burkitt's lymphoma and NK/T cell lymphoma as well as carcinomas such as nasopharyngeal carcinoma (NPC) and EBV-associated gastric carcinoma (EBV-GC). However, to date no available prophylactic vaccine was launched to the market for clinical use.

**Methods**: To develop a novel vaccine candidate to prevent EBV infection and diseases, we designed chimeric virus-like particles (VLPs) based on the hepatitis B core antigen (HBc149). Various VLPs were engineered to present combinations of three peptides derived from the receptor binding domain of EBV gp350. All the chimeric virus-like particles were injected into Balb/C mice for immunogenicity evaluation. Neutralizing titer of mice sera were detected using an *in vitro* cell model.

**Results**: All chimeric HBc149 proteins self-assembled into VLPs with gp350 epitopes displayed on the surface of spherical particles. Interestingly, the different orders of the three epitopes in the chimeric proteins induced different immune responses in mice. Two constructs (149-3A and 149-3B) induced high serum titer against the receptor-binding domain of gp350. Most importantly, these two VLPs elicited neutralizing antibodies in immunized mice, which efficiently blocked EBV infection in cell culture. Competition analysis showed that sera from these mice contained antibodies to a major neutralizing epitope recognized by the strong neutralizing mAb 72A1.

**Conclusion**: Our data demonstrate that HBc149 chimeric VLPs provide a valuable platform to present EBV gp350 antigens and offer a robust basis for the development of peptide-based candidate vaccines against EBV.

## Introduction

Epstein-Barr virus (EBV), a widespread human γ-herpesvirus, causes persistent infection in more than 95% of the world population 1, 2. Primary EBV infection is usually asymptomatic and often occurs during childhood. EBV is the major cause of infectious mononucleosis (IM) and is also tightly linked to various malignancies including Hodgkin's lymphoma (HL), Burkitt's lymphoma (BL), NK/T cell lymphoma, nasopharyngeal carcinoma (NPC) and gastric carcinoma (GC) 3. Immunocompromised individuals such as transplant recipients and AIDS patients are at increased risk of developing EBV-associated malignancies 4-7.

It is urgent to develop an effective vaccine to prevent EBV infection 8, 9. However, to date, there is no approved vaccine against EBV infection and EBV-associated diseases. EBV encodes many envelope glycoproteins. The most abundant glycoprotein on the virion surface, gp350 has been one of the most studied targets for development of a prophylactic subunit vaccine to neutralize infection of B cells. In response to natural EBV infection, gp350 is the major immunogen to induce a neutralizing antibody response in human sera, which protects B cells against infection 10, 11. Immunization with antigens comprising glycoproteins from the viral fusion apparatus (gH/gL, gH/gL/gp42 and gB) have elicited robust antibody response that have the advantage of neutralizing infection of B cells as well as epithelial cells 12-15 . Thus, the fusion apparatus glycoproteins have become components of choice in the design of prophylactic vaccines. Finally, antibodies against EBV BMRF2 prevented virus attachment and and entry into epithelial cells 16. The need for efficient protection of the two EBV target cell types in vivo, B cells and epithelial cells, will likely require immunization with combinations of viral glycoprotein antigens using common platform such as nanoparticles or VLPs. Recombinant gp350/220 proteins expressed in *E.coli* and insect cells were used to define the region reacting with the virus capsid antigen (VCA)-positive human sera 17. MAbs against gp350/220 showed neutralizing activity to prevent EBV infection 18, 19. A representative mouse monoclonal antibody (mAb) 72A1, effectively blocked EBV infection of B cells 20, 21. Moreover, mAb 72A1 directly bound to an epitope on the glycan-free surface which was identified as the receptor binding domain (RBD) of gp350 22, 23. The interaction between gp350 and the complement receptor type2 (CR2/CD21) on B lymphocytes is needed to trigger infection. The RBD is located at the N-terminus of gp350, and soluble proteins containing the RBD (i.e. gp350^FL^, gp350^1-470^) could block EBV infection of B cells. Overall, these data show the importance of the RBD of gp350 as a target for neutralization and support the use of the gp350 RBD as a promising subunit vaccine candidate against EBV.

Several approaches have been tested to develop an efficient vaccine candidate based on gp350 9. Soluble forms of the gp350 ectodomain expressed in CHO cells exhibited native conformation, bound the receptor CR2 and were recognized by several specific mAbs. The monomeric form of gp350 with native conformation induced high serum antibody titer that effectively neutralized EBV *in vitro* 24. A truncated version of gp350 (gp350^1-443^), which contained the RBD, was produced from *Pichia pastoris* and induced high levels of specific antibodies and a strong T cell response in mice 25. In addition, multimerization of gp350 antigens was shown to improve efficiency of vaccine candidates. Cui *et al*. demonstrated that a tetrameric gp350^1-470^ antigen induced higher neutralizing titer 26. Recently, we showed that the dimeric antigen gp350ECD123 (amino acid 1-425, referring to the first three N-terminal domains) fused with immunoglobulin Fc fragments elicited a stronger humoral immune response in mice compared to the monomeric counterpart 27. When expressed on self-assembling nanoparticles based on the ferritin protein, the gp350 RBD induced potent and durable neutralizing antibodies that prevented EBV infection in challenged mice 15. VLPs based on Newcastle disease virus (NDV) matrix and nucleocapsid were engineered by fusing the gp350 ectodomain or RBD with the NDV F protein. These NDV-VLPs bound to CD21 and CD35 and elicited a long-lasting neutralizing antibody response in mice 28. A cell line, HEK293-VII+, was engineered to produce EBV-derived VLP lacking viral DNA but presenting full-length gp350, gp140 and gp125 on their surface. These VLPs induced strong humoral response in a murine model *in vivo* and stimulated CD8+ and CD4+ T cell responses *in vitro* 29.

Furthermore, nonhuman primates immunized with different gp350-based vaccine candidates were protected from EBV-induced lymphomas when challenged with native virus. Immunization with purified membrane containing gp350 (previously named gp340) or with recombinant gp350 could protect tamarins from EBV-induced diseases 30-33. Recombinant vaccinia virus and replication defective adenovirus expressing gp340 effectively protected cottontop tamarins from EBV-induced malignant lymphomas 34-36.

Recently, gp350-based candidate vaccines have been evaluated in human clinical trials. A vaccinia virus vector-based vaccine was tested in infants and all developed neutralizing antibodies 37. In a phase I and I/II clinical trial, a recombinant subunit gp350-based vaccine elicited gp350-specific neutralizing antibodies 38. In addition, recombinant gp350 adjuvanted with aluminum hydroxide and 3-O-desacy-4'-monophosphory lipid A (AS04) efficiently prevented IM but did not prevent EBV acquisition in a cohort of seronegative individuals 39. A gp350-based vaccine was also tested in 13 children patients with chronic kidney disease. Neutralizing antibodies were detected in four recipients but the immune response declined rapidly 40. These attempts highlight the benefits of gp350 immunization but also the needs to improve immunogenicity and promote a long lasting immune response.

Epitope peptides are considered to be valuable vaccine candidates, especially for conserved neutralizing epitopes 41, 42. Several peptides derived from the gp350 RBD efficiently blocked CR2 binding to EBV or to recombinant gp350 and could bind to CR2-positive B cells. Additionally, antibodies directed against these peptides inhibited EBV binding to B cells 43, 44.

Compared to full-length or truncated protein antigens, linear peptides do not present conformation-dependent epitopes. Although peptides allow targeting of precise functional domain, their conformational limitations may restrict the immunogenicity of peptide-based vaccines. The immunogenicity of epitope peptides with low molecular weights can be enhanced by multimerization 16 or by presentation of the peptides on the surface of recombinant VLPs. Here we used chimeric VLPs formed by the C-terminally truncated Hepatitis B virus core (HBc) protein (149 aa), which have been considered as an ideal delivery vehicle for peptides derived from HIV, HCV, EV71, CA16, VZV, HEV, ZIKV and dengue virus 45-51. Practically, chimeric HBc proteins produced in *E.coli* self-assemble and display foreign epitope peptides on the surface of VLPs 52, 53. The region between amino acid 78 and 82 (MIR) is an ideal insertion site because it is surface accessible and not required for HBc self-assembly 54, 55. In this study, we selected three peptides named P1 (aa 16-29), P2 (aa 142-161) and P3 (aa 282-301) from the gp350 RBD and used the truncated HBc149 as an immune carrier. The three peptides were inserted into the MIR of HBc149 in different tandem order combinations. All five constructs yielded well-formed spherical particles. Interestingly, different arrangements of the three epitopes greatly influenced the humoral response of immunized mice. Two configurations, 149-3A (P1P2P3) and 149-3B (P1P3P2) elicited high antibody titers against gp350ECD123 (corresponding to gp350^1-425^), while other combinations were poorly immunogenic. In addition, sera collected from 149-3A and 149-3B immunized mice showed high competitive activity with a neutralizing mAb 72A1, thereby indicating the presence of Abs against a major neutralizing epitope of gp350. More importantly, sera from 149-3A and 149-3B-immunized mice neutralized EBV infection of cells *in vitro*. Altogether these data support the use of HBc-VLPs as useful vectors for gp350 peptides to induce anti-EBV neutralizing antibodies.

## Materials and Methods

### Plasmids Construction for Expression of Recombinant HBc149-based Fusion Proteins

Plasmid pET-28a-HBc149 was constructed as previously reported 56. Briefly, the encoding sequence of HBc149 was inserted into the vector pET-28a between the restriction endonuclease sites of *Nde I* and *Hind III*. Amino-acids 79-81 were replaced by GGGGSGGGGT-GS-EF-GGGGSGGGGS in which GS was coded by a *BamH I* site and EF was coded by an *EcoR I* site. The combination sequences coding for the gp350 peptides were synthesized (Beijing Ruibio Biotech Co., Ltd) and inserted into the linearized vector. The five combinations were: P1-L-P2-L-P3 (3A), where L represents the G_4_SG_4_S linker, P1-L-P3-L-P2 (3B), P2-L-P1-L-P3 (3C), P2-L-P3-L-P1 (3D) and P3-L-P2-L-P1 (3E). The fusion clones were confirmed by sequencing and the five constructions were named 149-3A, 149-3B, 149-3C, 149-3D and 149-3E respectively. The wide-type HBc149 vector was used as a control.

### Protein Expression and Purification

Plasmids coding the various constructs were transformed into BL21 (DE3) competent bacteria. Positive clones were selected and amplified in liquid cultures. Then, 5 ml of fresh overnight cultures were inoculated into 500 ml fresh LB medium in the presence of 100 mg/L kanamycin at 37°C. Proteins production was induced by adding IPTG to a final concentration of 0.5 mM at 30°C for 6-8 h when OD_600_=0.8.

The harvested bacterial pellets were resuspended in PBS (pH 7.4). After ultrasonication and high speed centrifugation (25000 x g, 30 min), the supernatants were collected and precipitated using 30% saturated ammonium sulfate. The precipitated material was resuspended in PBS and dialyzed against PBS. The proteins were further purified by gel filtration using superpose 6 increase 10/300 GL columns (GE). The concentration of the target proteins were determined using BCA assay. The purified proteins were stored at -80°C prior to use.

### SDS-PAGE and Western blotting (WB)

The protein samples were subjected to 12% SDS-PAGE gels for 1 h at 160 V and the total proteins were visualized by coomassie brilliant blue staining.

For WB, after SDS-PAGE, proteins were transferred onto polyvinylidene fluoride membranes (Millipore). The membranes were blocked with 5% skim milk in TBST buffer (25 mM Tris, 250 mM NaCl, 0.1% Triton-20, pH 7.4) for 2 h at room temperature. To detect the HBc149 antigen, mAb 11H10 (gift from Prof. Xia of Xiamen University) was used (1:2,000 dilution). For detection of gp350 epitopes, an antiserum prepared in our lab was used (1:1,000 dilution). The membranes were inoculated with diluted antibodies at 4°C overnight. After three washes with TBST, membranes were incubated with a secondary goat anti-mouse antibody (1:10,000, Promega) conjugated with horseradish peroxidase (HRP) for 1 h at room temperature. ECL substrate was used for detection (Advansta).

### Transmission Electron Microscopy (TEM)

The recombinant proteins were analyzed by negative staining electron microscopy. Briefly, protein samples were diluted to 0.5 mg/ml and applied to 200-mesh carbon-coated copper grids for 5 min. Excessive solution was removed, grids were washed twice with double distilled water and then immediately negatively stained for 30 s with freshly filtered 2% phosphotungstic acid (pH 6.4). Grids were examined with a FEI Tecnai T12 TEM (FEI, USA) at an accelerating voltage of 120 kV and photographed at a magnification of 25,000 fold.

### High Performance Size Exclusion Chromatography (HPSEC)

All purified proteins were analyzed using a 1120 Compact LC HPLC system (Agilent Technologies; Santa Clara, CA) and separately with a TSK Gel PW5000xl 7.8 mm × 300 mm column (TOSOH, Japan); columns were pre-equilibrated in PBS. The column flow rate and protein signal detection for the SEC analysis were 0.5 ml/min and 280 nm.

### Indirect Enzyme-Linked Immunosorbent Assay (ELISA)

Purified gp350^1-425^-His was coated on 96-well microplates (Corning) (100 ng/well in PBS) for 2 h at 37°C. The plates were washed once and then blocked with blocking buffer (PBS pH 7.4, containing 2% gelatin, 0.5% casein and 0.1% ProClin 300) overnight at 4°C. Then, 5-fold serial dilutions of sera were added to the plates and incubated for 1 h at 37°C. The plates were washed 5 times and incubated for 30 min at 37°C with 100 μl of horseradish peroxidase (HRP)-conjugated goat anti-mouse IgG (Promega) (1:20,000 dilution). Signals were developed using EL-TMB kit (Sangon Biotech). Absorbance was measured at 450 nm using a microplate reader (Molecular Devices). The cutoff value was set to 0.1 which was determined by the OD_450_ value of preimmune sera.

### Competitive Enzyme-Linked Immunosorbent Assay (ELISA)

MAb 72A1 (ATCC cell line ID: HB168) conjugated with HRP was used in this assay. First, 2-fold dilutions of 72A1-HRP were used to determine the OD450 for binding to gp350^1-425^-His coated on the plates (100 ng/well). A 1.0 value for OD450 was arbitrarily selected for competition assays and corresponds to a 1:25,600 dilution. Second, in competition assay, 2-fold serially diluted sera (starting from 1:5) were added to the gp350^1-425^-His coated plates and incubated for 1 h at 37°C. Then 72A1-HRP (1:25,600 dilution) was added to the plates after 5 TBST wash and incubated for 30 min at 37°C. Bound HRP activity was detected using the EL-TMB kit (Sangon Biotech). The competitive ability of the sera samples against 72A1 was calculated using the following equation: Percentage of inhibition %=[OD_(-serum/+72A1)_-OD_(+serum/+72A1)_]/ OD_(-serum/+72A1)_ x 100.

### Mouse Immunization Assay

Five female special pathogen free (SPF) female BALB/C mice (6-8 weeks) per group were immunized subcutaneously (s.c.). The proteins were formulated with aluminum hydroxide so that each dose contained 20 μg purified proteins. One primary injection dose and two booster doses were given at two week intervals (week 0, 2, 4). The immunized mice were bled at weeks 0 (preimmune), 1, 2, 3, 4, 5, 6, 8, 10 and14 for serological tests. All collected sera were stored at -20°C prior to use.

### Infection Blocking Assay

The EBV infection assay was performed as previously reported 20. AKATA-EBV-GFP was produced in CNE2-EBV cells 57. Representative mAb 72A1 or 2-fold serial diluted sera (starting from 1:2) were incubated with 100 μl virus stock in 1.5 ml tubes for 2 h at 37°C. Subsequently, the mixture of mAb or sera with EBV were added to10^5^ AKATA negative cells (no latent EBV) at 37°C for 3 h. After incubation, the cells were pelleted by centrifugation and washed once using PBS before being cultured in RPM1640 with 10% FBS in 24-well plates for 48 h. The cells were then collected and washed once with PBS. The EBV infection rate was determined by measuring the production of GFP expressing cells by flow cytometry. In this assay, uninfected cells were used as negative controls and AKATA negative cells incubated with EBV in the absence of antibodies were used as positive controls.

### Homology Modeling

The crystal structures of gp350ECD123 (PDB no. 2H6O) and HBc149 (PDB no. 1QGT) were used as templates for homology modeling of the chimeric monomers. After sequence alignment with the respective chimeric sequences, the initial 3D models were generated using the Homology module of Discovery Studio 2.5 program (Accelrys). HBc149 (PDB no. 1QGT) VLP template was utilized to generate the complete chimeric VLP models. Stepwise minimizations were subsequently implemented to the peptides insertion site of each model to achieve thermodynamically favored conformations.

### Statistics

All statistical analyses were carried out with GraphPad Prism version 5. p-Values were generated by one-way ANOVA analysis. p-Values of ≤0.05 were considered to be statistically significant.

## Results

### Rational molecular design of fusion proteins

**Fig 1:**
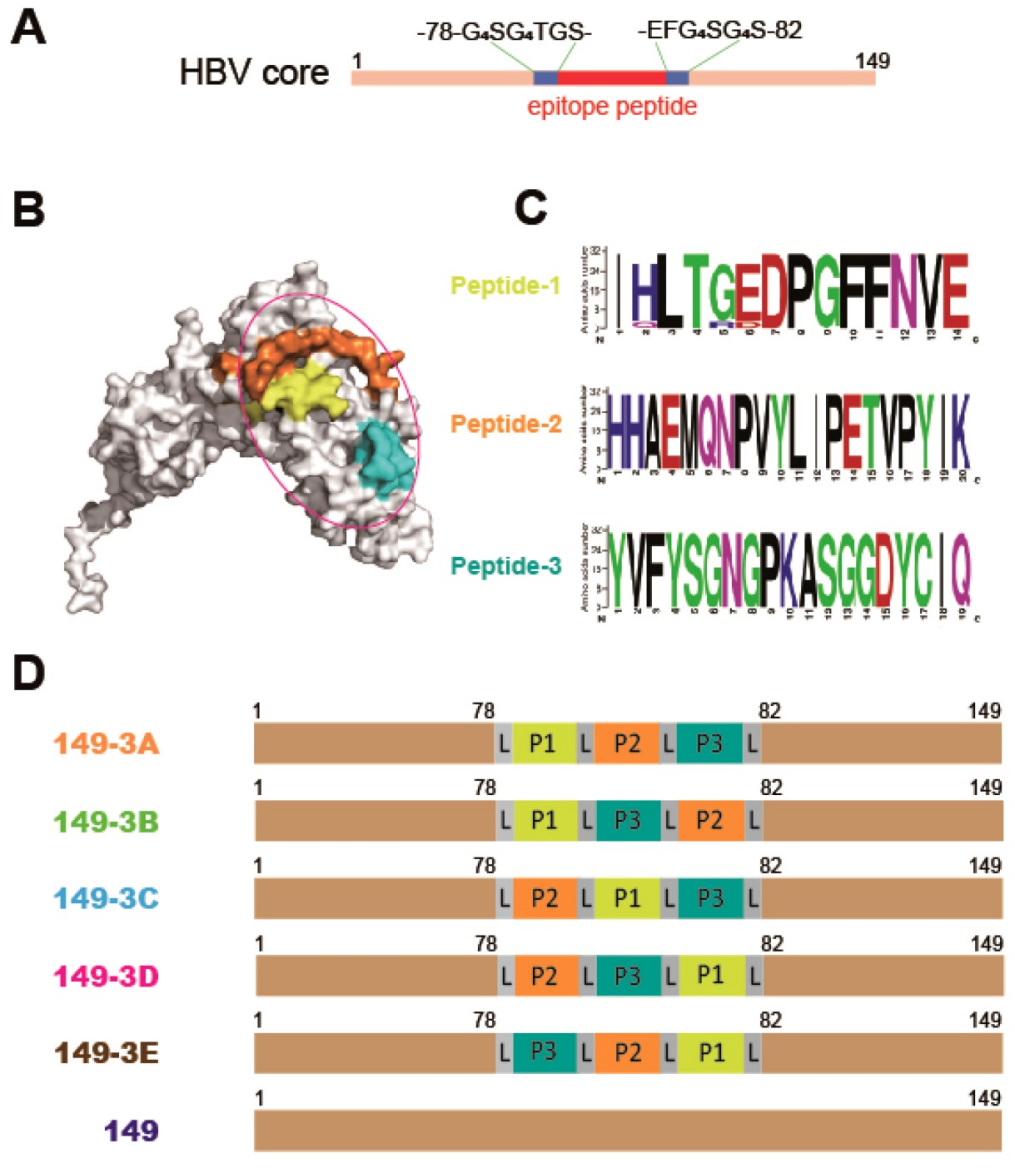
** Schematic diagram of the rational design and peptides combination vaccine candidates. (A)** Truncated HBV core protein (HBc149) was engineered for convenient presentation of foreign epitopes. **(B)** The crystal structure of the gp350 N-terminal (PDB no. 2H6O) is shown as a surface model. The selected three peptides are colored with yellow (peptide 1, P1), brown (peptide 2, P2) and cyan (peptide 3, P3). The receptor binding domain is circled in purple.** (C)** Sequence alignment of P1, P2 and P3 peptides using 32 independent sequences from genebank. The sequence logos were generated using weblogo (http://weblogo.berkeley.edu/). Numbers, on the y-axis, represent the total sequences used in this alignment. **(D)** Schematic representation of the different constructs. Epitope peptides P1, P2, P3 were inserted into the modified HBc149 vector in different orders linked with GGGGSGGGGS linker (L).

In this study, a C-terminal truncated HBc protein (aa 1-149, HBc149) was used as a carrier protein to enhance the immunogenicity of gp350 RBD epitope peptides. The HBc149 protein was modified and amino acids 79-81 were substituted with GGGGSGGGGTGSEFGGGGSGGGGS to provide an insertion site flanked by flexible G_4_SG_4_S linkers (Fig. 1A). This allows for the insertion of foreign epitope peptides in a site exposed at the surface of VLPs. The crystal structure of gp350ECD123 has been determined and the receptor-binding domain was identified by mutagenesis 22. Three linear peptides that form part of the RBD at the surface of gp350 were selected (Fig. 1B). Sequences of these peptides was aligned using MEGA 5.05 and Weblogo programs. The alignments showed high levels of conservation of all three peptides among 32 EBV gp350 sequences from Genebank (Fig. 1C). We designed five different arrangements of three peptides (149-P1/P2/P3, 149-P1/P3/P2, 149-P2/P1/P3, 149-P2/P3/P1 and 149-P3/P2/P1) and the corresponding fusion proteins were named 149-3A, 149-3B, 149-3C, 149-3D and 149-3E respectively (Fig. 1D). The unmodified HBc149 (wild-type) and soluble gp350ECD123 27 were used as controls in subsequent assays.

### Expression and characterization of fusion proteins

**Fig 2:**
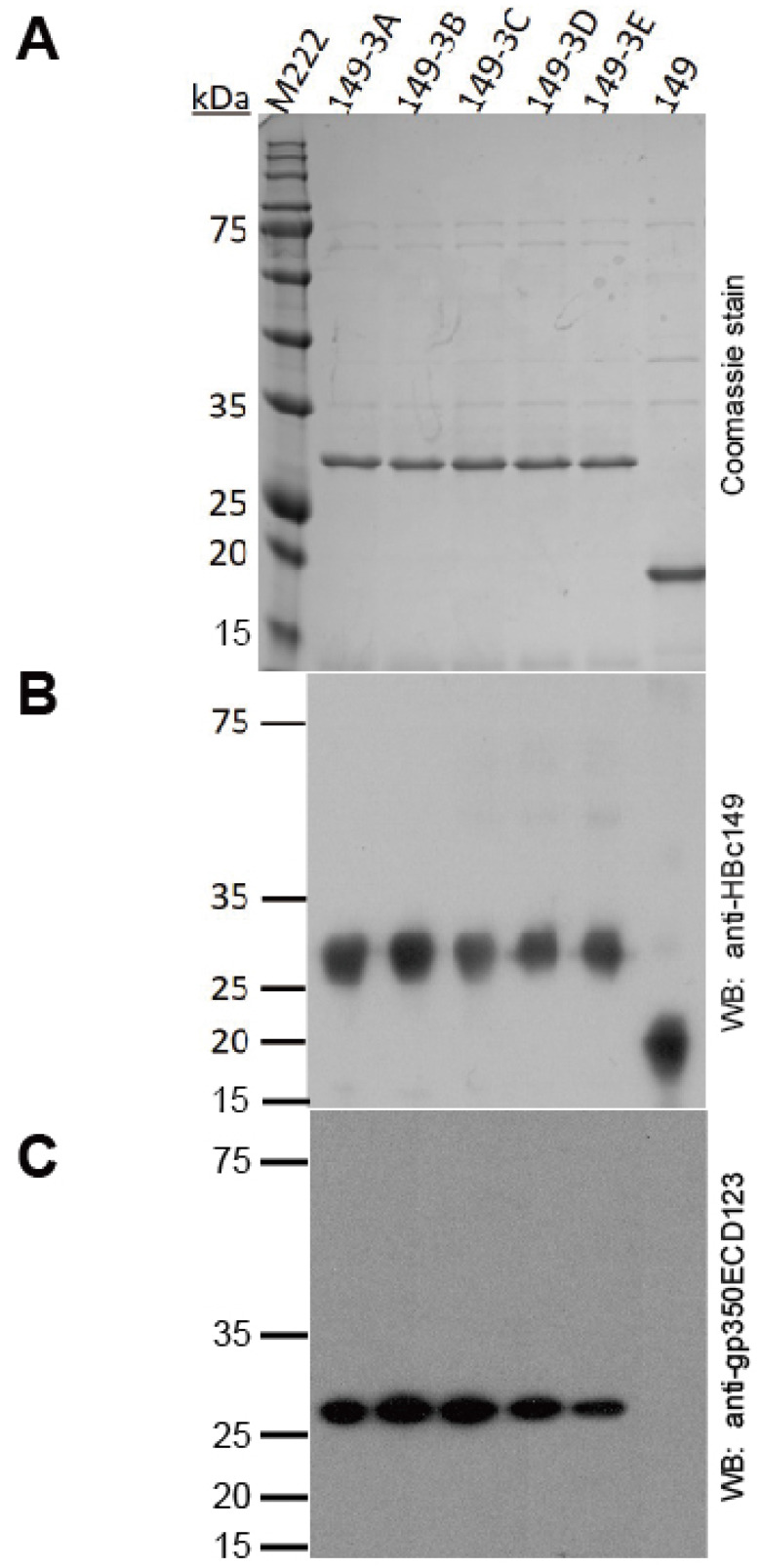
** Analysis of the *E.coli*-expressed HBc149 fusion proteins by SDS-PAGE. (A)** SDS-PAGE analysis of the purified fusion proteins stained by coomassie brilliant blue.** (B)** Western blot analysis of fusion proteins with anti-HBc149 mAb 11H10.** (C)** Western blot analysis of fusion proteins with serum anti-gp350ECD123.

All the fusion proteins were expressed in BL21 (DE3) bacteria. Analysis of HPSEC purified proteins by SDS-PAGE shows high purity and homogeneity of each product (Fig. 2A). In addition, mAb 11H10 against HBc149 and antisera against gp350ECD123 recognized the fusion proteins in WB assay (Fig.2B and 2C).

**Fig 3:**
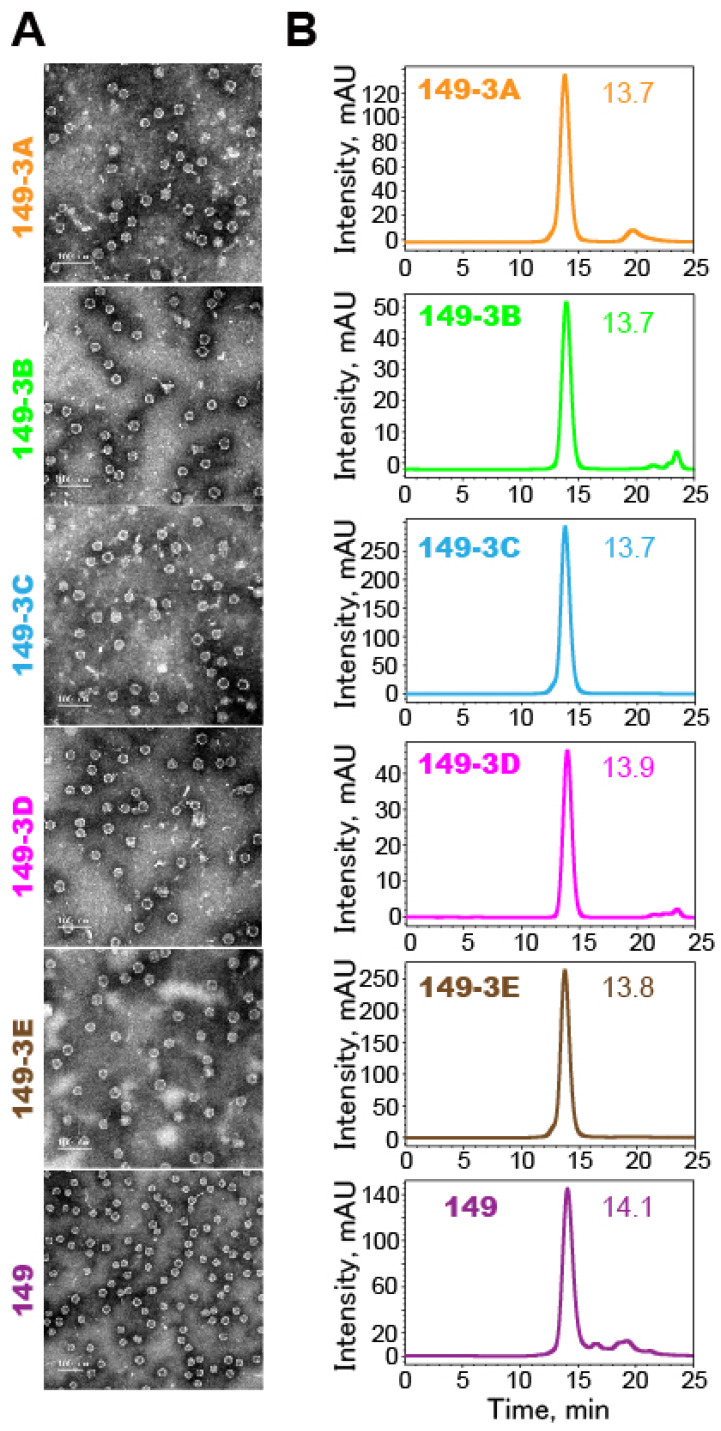
** Characterization of self-assembly of the fusion proteins into VLPs. (A)** TEM images of suspensions of VLPs (magnification: 25,000x). Scale bars represent 100 nm.** (B)** Analysis of VLPs by HPSEC. Elution profiles (arbitrary units over time) show that all preparations are highly homogenous. The peak elution time is indicated.

Particle profiles were analyzed using negative-stained TEM and HPSEC (Fig. 3). The results showed that all the fusion proteins self-assembled into particles of similar sizes and morphologies. The inserted peptides in various orders did not have a detectable effect on the folding and self-assembly of chimeric HBc149 VLPs.

### Immunogenicity of the chimeric VLPs

**Fig 4:**
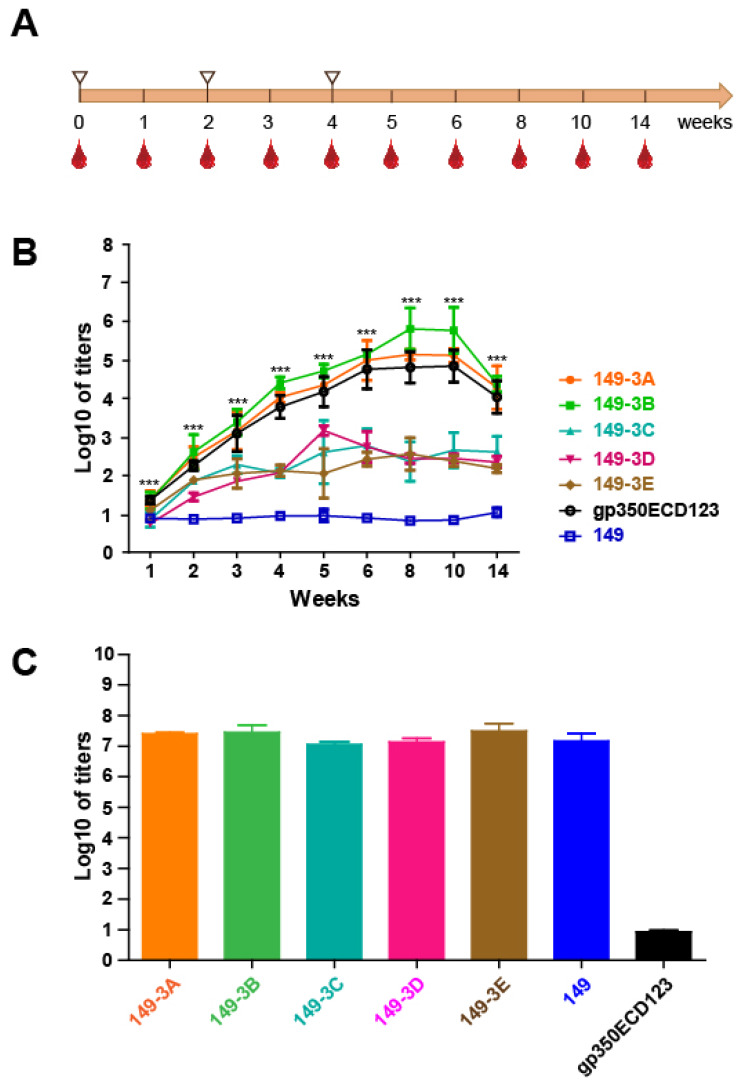
** Immunogenicity of the purified fusion proteins. (A)** Immunization procedure. Immunization (white triangles) and bleeding time points are shown (red drops).** (B)** Anti-gp350 titers determination by ELISA. gp350ECD123 was immobilized on 96-well plates and the diluted sera were added for titers determination. The OD450=0.1 was set as the cut-off value. Significance (*p≤0.05, **p≤0.01, ***p≤0.001) between 149-3A and 149-3B VLPs versus gp350ECD123 is shown. Both constructs 149-3A and 149-3B have the same level of significance above gp350ECD123. (**C**) Binding titers of serum antibodies to the vector (HBc149) collected after eight weeks. The error bars indicated the standard deviation in each group (n=5 mice).

To assess the immunogenicity of the chimeric VLP, BALB/c mice (n=5 per group) were inoculated three times at 2-week intervals with chimeric VLPs (Fig. 4A). The antigens were formulated with aluminum adjuvant using 20 μg proteins at each injection. Serum was collected over 14 weeks and antibody titer against soluble gp350ECD123 was determined by ELISA. VLPs 149-3A and 149-3B elicited higher antibody titer than soluble gp350ECD123 controls (Fig. 4B). The sera titer increased to about 5-log for 149-3B and 4.5-log for 149-3A in comparison with 4-log for gp350ECD123 at 1 week after the third injection (week 5). The peak titers of approximately 6-log for 149-3B and 5-log for 149-3A (4.5-log for gp350ECD123) were measured at 8 weeks, and the sera titer were maintained at 4.5-log for 149-3A and 149-3B for at least 14 weeks after the first immunization. The data demonstrated that selected gp350 peptides presented on 149-3B and 149-3A VLPs can induce higher anti-gp350 antibody titers than the monomeric gp350ECD123 protein (Fig. 4B).

Surprisingly, the other three fusion proteins, 149-3C, 149-3D and 149-3E did not induce comparable sera titer, suggesting that the order of gp350 peptides in HBc149 constructs is critical for immunogenicity of VLPs. To confirm that all VLPs were equally injected during immunization, the binding activity of immune sera to HBc149 was tested (Fig. 4C). There was no significant difference of antibody titers against HBc149 for the five fusion proteins and the HBc149 positive control (Fig. 4C). Because VLPs were generated in bacteria and gp350ECD123 was produced in insect cells, it could be possible that the immunogenicity of VLPs was artificially enhanced by an adjuvant effect due to contamination with high amounts of lipopolysaccharides (LPS). We therefore quantified the levels of endotoxin in the protein samples that were used for immunization. There was no difference in endotoxin content between samples of the various VLPs, and these levels were not different from levels in the insect cell-produces gp350ECD sample (Figure S1). These data indicate that an adjuvant effect of LPS endotoxin contaminants is not responsible for the difference in immunogenicity observed in our *in vivo* experiments. Overall, our data show that all groups received similar amounts of purified VLP antigens and that the difference of anti-gp350 titers observed between constructs is due to the presentation of peptides on VLPs.

### Chimeric VLPs (149-3A and 149-3B) induced potent production of neutralizing antibodies

**Fig 5:**
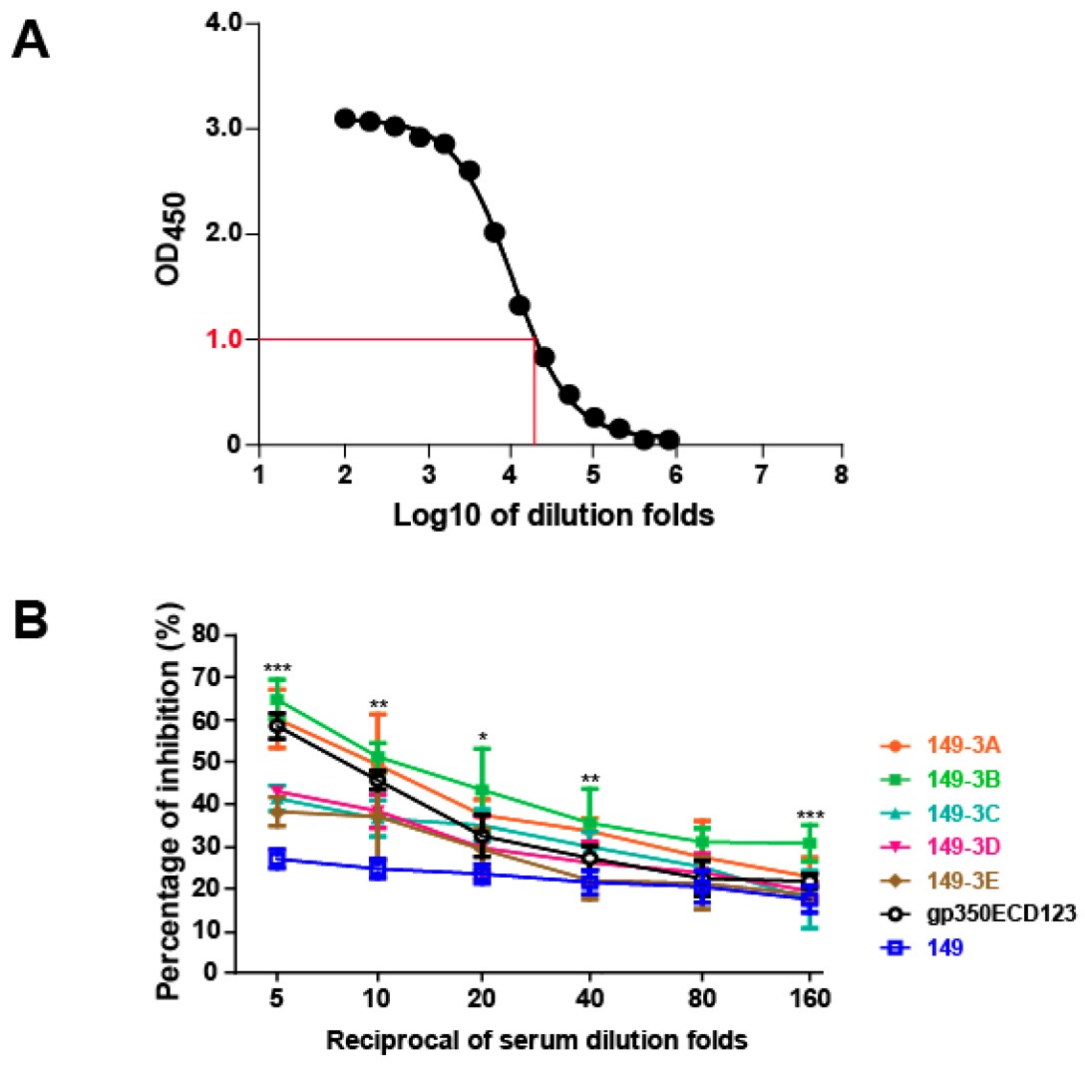
** Blocking the binding of the neutralizing mAb 72A1 to gp350ECD123 in a competition ELISA. (A)** Binding of mAb 72A1-HRP to immobilized gp350ECD123. Serial dilutions of antibody were used to determine the dilution factor corresponding OD450=1.0.** (B)** Binding of mAb 72A1-HRP to immobilized gp350ECD123 is competed by the indicated immune sera. Serial dilution of sera were applied to gp350ECD123 before the addition of mAb 72A1-HRP in this competition ELISA. Data are shown as % inhibition of mAb 72A1binding. High inhibition indicates the presence of competing antibodies in the tested sera. Significance (*p≤0.05, **p≤0.01, ***p≤0.001) is indicated for 149-3A and 149-3B VLPs compared to gp350ECD123. Both constructs 149-3A and 149-3B have the same level of significance above gp350ECD123.

To determine whether VLP's elicited antibodies against a major neutralizing epitope of gp350, immunized sera were evaluated using a competition ELISA 27. The mAb 72A1, is a strong neutralizing antibody against EBV infection into B cells that binds to the gp350 RBD 22, 23. To assess the presence of serum antibodies against the same neutralizing epitope, we used a competition ELISA against mAb 72A1. First, we established a binding curve for mAb 72A1-HRP to gp350ECD123 (Fig. 5A). The dilution fold of 1:25,600 for 72A1-HRP corresponds to a value of 1.0 OD450 and was selected for competition assays. In competition assays, serially diluted sera were used to inhibit binding of 72A1-HRP to gp350ECD123. A decreased binding of mAb 72A1-HRP indicates the presence of competing antibodies, presumably to the same epitope, in immune sera. The results indicated that sera from mice injected with 149-3A and 149-3B showed better competition compared to sera from mice immunized with gp350ECD123 (Fig. 5B). At 5-fold and 10-fold dilution, the sera inhibited more than 50% binding of 72A1-HRP. As expected, low titer sera from mice immunized with 149-3C, 3D and 3E did not efficiently compete with mAb 72A1 binding. Sera from mice immunized with control HBc149 alone show a constant background of inhibition, consistent with previous studies 27, 58. This competition assay demonstrates that peptides displayed on HBc-VLPs in defined orders elicit a potent antibody response against gp350. Furthermore, competition data showed that these antibodies detect epitopes which overlap with the major neutralizing epitope recognized by mAb 72A1.

**Fig 6:**
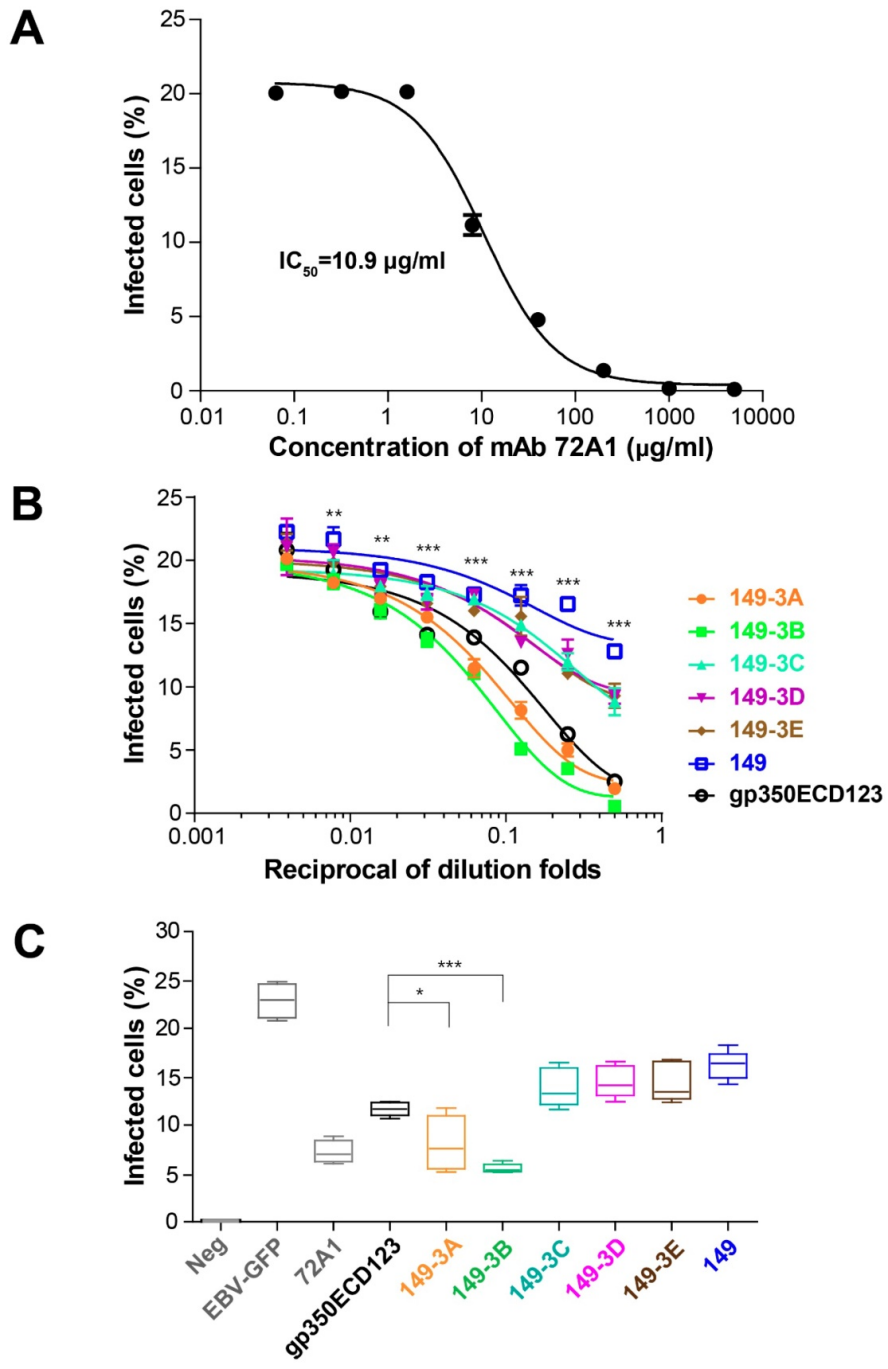
** Neutralizing of EBV infection. (A)** Neutralizing curve for mAb 72A1 against EBV-GFP infection of AKATA cells. M Ab 72A1 was used to validate the neutralization assay and its IC_50_ is indicated. (**B**) Neutralization of infection by immune sera. Serially diluted sera collected at week 8 were used to block EBV infection of AKATA cells. Significance (*p≤0.05, **p≤0.01, ***p≤0.001) is indicated for 149-3Aand 149-3B VLPs compared to gp350ECD123. Both constructs 149-3A and 149-3B have the same level of significance above gp350ECD123. (**C**) Sera collected at week 10 was diluted 10 fold to block EBV infection into AKATA cells (n=5). Significance (*p≤0.05, **p≤0.01, ***p≤0.001) between the indicated fusion proteins versus gp350ECD123 is shown. EBV-GFP show the level in the absence of inhibitors. Neg shows the background level in the absence of infection. 72A1 shows the level of inhibition by mAb 72A1 at 50 μg/ml.

To confirm that serum antibodies have a functional neutralizing activity, an *in vitro* neutralization assay was used to evaluate the efficiency of sera in blocking EBV infection of AKATA cells. To set up the neutralizing assay, mAb 72A1 was used as a positive control. In the absence of antibodies, about 20% of cells were infected by the EBV-GFP reporter virus. In this assay, the IC_50_ for mAb 72A1 was 10.9 μg/ml (Fig. 6A). Serially diluted sera collected at eight weeks were used. Sera from mice immunized with 149-3A and 149-3B showed stronger neutralizing efficiency compared to the sera raised against soluble gp350ECD123. At 2-fold, 4-fold and 8-fold dilutions, the sera raised against 149-3A and 149-3B showed over 50% neutralizing efficiency against EBV infection. The corresponding ID50 values are 13.43 and 18.93 for sera from animals immunized by 149-3A and 149-3B respectively (Table S1). (Reviewer 3, point 4) Other constructs only show limited neutralization, close to non-specific activity and their ID50 values could not be reliably determined.

To better quantify the efficacy of the different antigens, sera collected at ten weeks were tested in this neutralization assay at a 10-fold dilution. Here again, sera collected from mice immunized with 149-3A and 149-3B blocked EBV infection of AKATA cells most efficiently (Fig. 6C). Importantly, the neutralization titers induced by 149-3B and 149-3A were significantly higher than that of the other fusion proteins and the monomeric gp350ECD123. This monomer is partially glycosylated in insect cells and can elicit conformation-dependent antibodies. In contrast, the fusion proteins 149-3A and 149-3B that were expressed in bacteria and contained linear gp350 peptides that lack glycosylation sites, induced a more potent neutralization response.

In order to confirm that sera 149-3A and 149-3B detect gp350 in EBV producing cells, we performed IFA staining of AKATA-EBV stimulated cells. In this assay, the same staining pattern was observed for the mAb 72A1 and the two neutralizing sera (data not shown).

In summary, the designed fusion proteins formed chimeric VLPs with different peptides combinations. The specific arrangement of three gp350 peptides on 149-3A and 149-3B VLPs generated higher anti-gp350 antibody titers compared to the subunit gp350ECD123 antigen. The sera collected from 149-3A and 149-3B VLPs immunized mice shared overlapped epitopes with mAb72A1 and neutralized EBV infection of B cells.

## Discussion

Vaccination is proven to be safe and cost-effective way to protect against pathogen infections and relevant infectious diseases. To date, there is no prophylactic vaccine for clinical use against EBV. Different subunit vaccines have been developed based on dimeric, tetrameric and polymeric forms of gp350 which elicited potent neutralizing antibodies and cytokine responses 15, 26, 27. Here we considered peptide-based approach as an attractive alternative strategy to direct the antibody response against a neutralizing site of gp350. The N-terminus fragment gp350^1-425^ retains the complete binding activity with the receptor CR2. Therefore, peptides localized to this region are ideal candidates for a rational design of EBV vaccines.

In our study, we selected three peptides that were previously identified by structural modeling, functional tests, and mutagenesis. The peptide corresponding to the N-terminus of gp350 (aa 16-29, IHLTGEDPGFFNVE) binds to CR2, inhibits CR2 binding to immobilized EBV and blocks recombinant gp350 binding to B cells 43. The other two peptides (aa 142-161, HHAEMQNPVYLIPETVPYIK and aa 282-301, YVFYSGNGPKASGGDYCIQS) are involved in EBV binding to B cells and inhibit mAb 72A1 binding to EBV 44. In addition, sera collected from rabbits immunized with these peptides contained anti-peptide antibody titers between 6,400 and 51,200 and slightly lower antibody titer against EBV (between 3,200 and 25,600) 44. The functional importance of these regions was confirmed by mutagenesis. Key residues (Glu^21^, Asp^22^, Tyr^151^, Glu^155^, Ile^160^, Trp^162^, Asp^208^, Glu^210^ and Asp^296^) are located at the interface between gp350 and CR2 59. X-ray crystallography further identified three discontinuous peptides at the interface corresponding to Pro^158^Tyr^159^Ile^160^, Trp^162^Asp^163^Asn^164^ and Asp^208^Glu^210^ 22. All the findings paved the way for rational design of epitope peptides-based vaccine against EBV.

**Fig 7:**
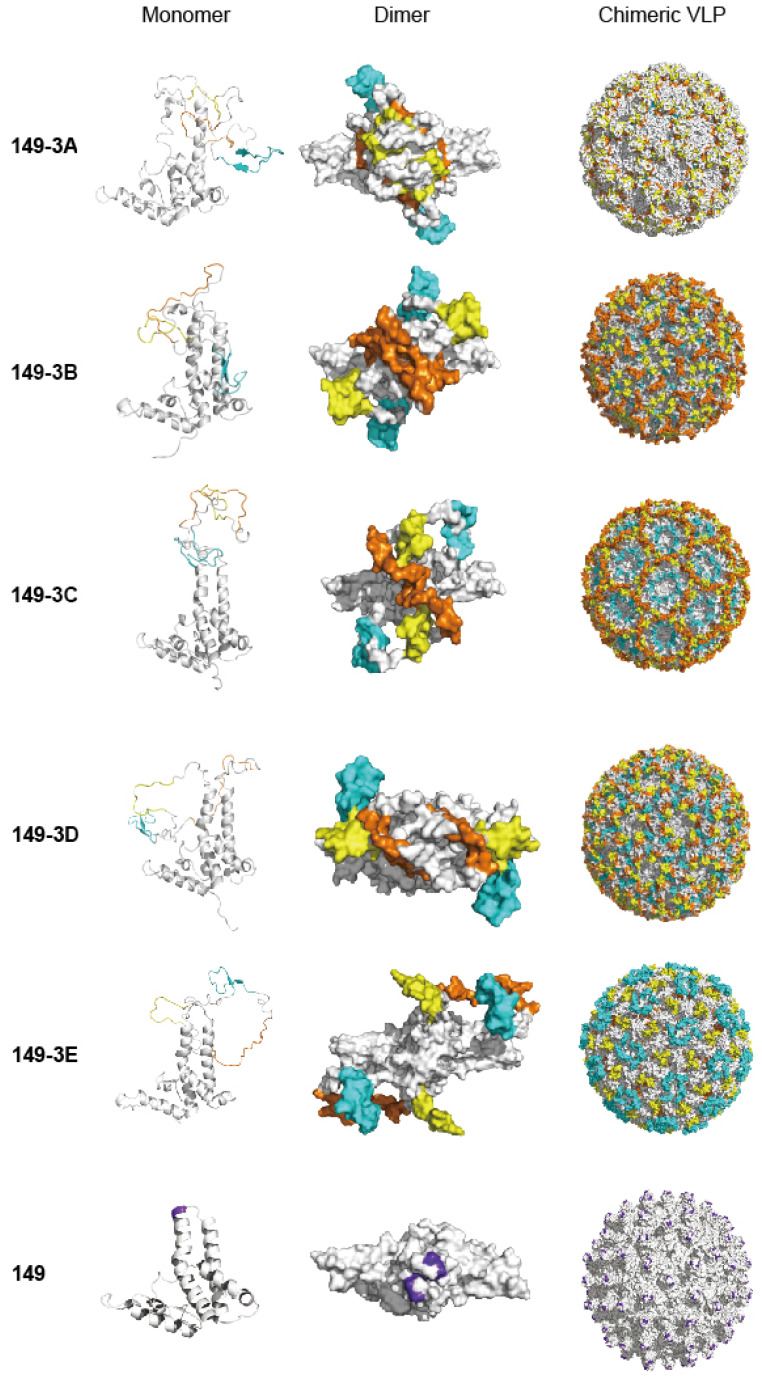
** Models of fusion proteins and chimeric VLPs corresponding to different constructs.** The left, middle and right panels show structural models for monomers (ribbon), dimers (surface) and chimeric VLPs (surface) of fusion proteins. Homology structural modeling of the recombinant fusion proteins and chimeric VLPs were implemented using MODELER module of Accelrys Discovery Studio 2.5. All illustrative models were prepared using PyMol. The three peptides are colored with yellow (peptide 1, P1), brown (peptide 2, P2) and cyan (peptide 3, P3). The peptide insertion site on HBc149 is shown in purple on the wild-type protein and VLP.

Often, one peptide epitope may not be sufficient to induce strong enough immune responses against viral infection. In this study, three epitope peptides were simultaneously displayed on the surface of chimeric VLP with 'G_4_SG_4_S' as a linker between them. Our study demonstrated the potential of the chimeric 149-3B and 149-3A combinations to generate immunogenic VLPs. Interestingly, for 149-3C, 149-3D and149-3E constructs, the orders of these epitopes affected the immunogenicity and the neutralizing activity of sera, but did not appear to influence assembly of the chimeric VLPs. Figure 7 shows structural models of the different fusion proteins and VLPs. Interestingly, the presentation of the peptides varies considerably between VLPs. Since the peptides are arranged in the same linear fashion but in different order (Fig. 1D), the structural variability is caused by the composition and folding of each peptide and the flexibility that each combination allows. The models of 149-3A and 149-3B suggest more flexible conformations of the peptides compared to 149-3C, 3D and 3E. This may allow 149-3A and 149-3B VLPs to generate stronger neutralizing antibodies against EBV infection.

Soluble monomeric gp350 is a relatively poor antigen, which immunogenicity is increased by about 20-fold through tetramerization 26, 60. We also previously showed increased immunogenicity through dimerization of gp350ECD123 27. Several other approaches with full-length or truncated gp350 showed superior immunogenicity and ability to induce neutralizing antibodies 60. Peptide antigens do not elicit antibodies against conformation-dependent epitopes, thus linear peptides often have reduced immunogenicity compared to proteins. Despite this intrinsic shortcoming, our selection of three peptides from a functional site on gp350 induced a better neutralization response than monomeric gp350ECD123. Nevertheless, our data reveal some limitation of the use of peptides, notably the effect of the positioning of the peptides on HBc-149 VLPs. Although peptide antigens offer the advantage of combining several targeted epitopes, the presentation of peptide antigen in VLPs need further improvement to reach the immunogenicity level of protein multimers.

Many factors can affect the success of peptide-based vaccines, our data highlight the importance of the order of epitopes when designing vaccines based on multiple epitopes. In addition to peptide display, VLPs, may affect epitope immunogenicity due to their own physical properties (e.g. posttranslational modifications, aggregation, solubility, intrinsic immunogenicity). Our data show that in the presence of aluminum hydroxide adjuvant, VLPs with peptide antigens performed better than the soluble protein antigen in eliciting neutralizing antibodies. Different adjuvants could help to further enhance the immunogenicity of chimeric VLPs 49.

In previous studies, gp350 was used as the major immunogen for vaccine development, although it is only effective in preventing infection of B cells. However, EBV encodes at least thirteen envelope glycoproteins that could be considered in vaccine design 61, 62. In particular, glycoproteins involved in the viral fusion apparatus (gL/gH, gp42 and gB) have become very promising targets for the development of prophylactic vaccines. Immunization with nanoparticles or multimeric forms of these glyproteins elicited robust neutralizing antibodies able to protect B cells as well as epithelial cells 13-15, 60. Indeed, potent neutralizing epitopes were localized on gH/gL, gB and gp42 63, 64. Antibodies raised against three epitope peptides derived from gH could inhibit the interaction between EBV and cord blood lymphocytes 63. Additional peptide epitopes identified *in silico* and in functional assays also warrant further functional investigation 64-67. Several CTL epitopes were identified in gp350 and, interestingly, among the peptides that bound HLA-A2, the LIPETVPYI peptide is contained within peptide P2 used in the present study 65. However this peptide failed to efficiently stimulate CTL effectors from IM donors *ex vivo* 65. In that study, a more distant gp350 peptide (VLQWASLAV, aa 863-871) was able to induce a functional CTL response *ex vivo* and in HLA-A2 transgenic mice, which became protected against a gp350-expressing recombinant vaccinia virus 65. Linear CD4 T-cell epitopes were also identified in gp350 (incl. aa 65-75, FGQLTPHTKAV and aa 163-183, DNCNSTNITAVVRAQGLDVTL) 66. Therefore, future designs of peptide vaccines will successfully include various epitopes from different antigens and integrate B and T cell epitopes into one antigenic formulation 9, 60. VLPs based on HBc149 provide an appropriate platform to engineer complex peptide-based epitopes. Our study opens the possibility to develop pluripotent vaccines by combining HBc149-derived VLPs engineered to express B and T cell peptide epitopes from different EBV glycoproteins.

This work provides encouraging results towards the design of novel EBV vaccine candidates on the basis of HBc protein mosaic VLPs that display epitope peptides derived from gp350. More generally, our data provide proof of principle for EBV vaccine design combining multiple antigenic epitopes on HBc149 VLPs. A multi-peptide strategy that includes epitopes from the fusion machinery (gL, gH, gp42 and gB) in addition to gp350 will further enhance the efficacy of VLPs as vaccines against EBV.

## Conclusion

Three epitopes among different strains derived from the receptor binding domain of EBV envelope protein gp350 were combined to be displayed on the surface of chimeric HBc149 VLPs. These well-formed spherical bionanoparticles were characterized by a combination of physico-chemical methods. Among these five VLPs, 149-3A and 149-3B showed higher immunogenicity compared to purified gp350ECD123. More importantly, these two particles induced stronger neutralizing antibodies in mice to block EBV infection in a cell model. Therefore, different combinations of epitopes could affect the overall immune response. Consequently, HBc149-based chimeric VLPs provided an effective platform for the development of novel peptide-based candidate vaccines against EBV.

### Ethics Statement

All experiments involving mice were approved by the Institutional Animal Care and Use Committee at the Sun Yat-sen University Cancer Center, and the animals were cared for in accordance with the institutional guidelines. All the mice were purchased from Beijing Vital River Laboratory Animal Technology Co., Ltd. (the joint venture of Charles River Laboratories in China).

## Supplementary Material

Supplementary materials and methods, figure and table.
