# Supplementary material for: A novel vaccine candidate based on chimeric virus-like particle displaying multiple conserved epitope peptides induced neutralizing antibodies against EBV infection
Source: Theranostics. 2020 Apr 27;10(13):5704–18. doi: 10.7150/thno.42494 (PMC7255000; doi:10.7150/thno.42494)
Supplement: Supplementary file 1 — Supplementary materials and methods, figure and table. [file thnov10p5704s1.pdf]

Supplementary Materials

Materials and Methods

The endotoxin level of each protein sample was measured by the toxinsensor™ chromogenic limulus amebocyte lysate (LAL) endotoxin assay kit according to the manufacturer’s instructions (GenScript, NewJersey, USA).

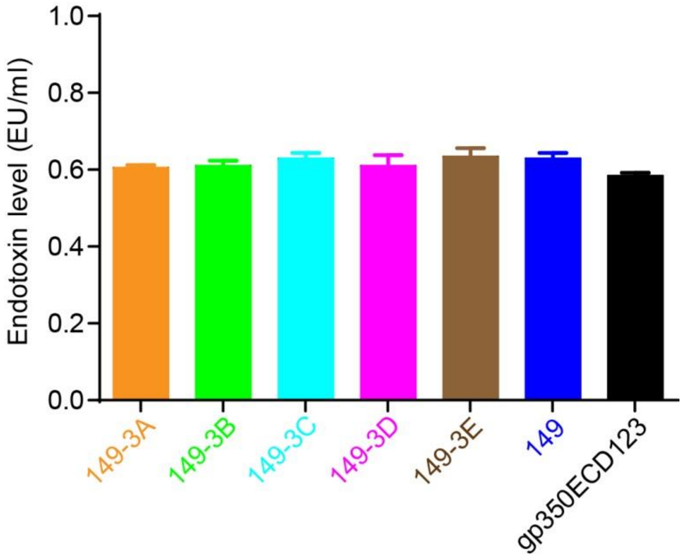

Figure S1 Detection of endotoxin level of different proteins used in immunization assay.

Table S1 Calculation of ID50 values for the serum raised by different proteins

Proteins	149-3A	149-3B	149-3C	149-3D	149-3E	149	gp350ECD123
ID50[1]	13.43	18.93	ND[2]	ND	ND	ND	9.91

[1] Inhibition doses calculated based on data from figure 6B

[2] ND indicates the ID50 value can not be calculated.
